# Net Survival in Survival Analyses for Patients with Cancer: A Scoping Review

**DOI:** 10.3390/cancers14143304

**Published:** 2022-07-06

**Authors:** Camila Macedo Lima Nagamine, Bárbara Niegia Garcia de Goulart, Patrícia Klarmann Ziegelmann

**Affiliations:** 1 Post-Graduate Program in Epidemiology, School of Medicine, Federal University of Rio Grande do Sul, Rua Ramiro Barcelos, 2400, Bairro Santa Cecilia, Porto Alegre 90035-002, Brazil; bngoulart@gmail.com (B.N.G.d.G.); patricia.ziegelmann@ufrgs.br (P.K.Z.); 2 Department of Exact and Technological Sciences, State University of Southwest Bahia, Estrada Bem Querer, Km-04-3293, 3391-Campus de Candeias–Vitória da Conquista, Bahia 45083-900, Brazil

**Keywords:** cancer survival, net survival, cancer registries, epidemiology, Pohar Perme Estimator

## Abstract

**Simple Summary:**

The Pohar Perme Estimator (PPE) is the gold standard for estimating net survival, however, few studies in the field of oncology have used this estimator. The PPE was identified as an important epidemiological indicator, which is easy to implement, produces unbiased estimates of net survival and allows comparison of survival between different populations. This scope review aims to clarify the potential benefits of the Pohar Perme Estimator for calculating the net survival of patients diagnosed with cancer and the justifications presented in the literature regarding the use, approach and application of the method. With this review, we recognize the importance of PPE in the field of oncology and we hope that it will be more used in the analysis of net survival, aiming to establish control strategies and improve the survival of these patients.

**Abstract:**

Population-based net survival is an important tool for assessing prognostic advances. The unbiased Pohar Perme Estimator (PPE) was suggested in 2012 and soon established itself as the gold standard for estimating net survival. This scoping review aims to know in which context this estimator is being used in the oncology area, what the authors point out as a justification for its use, and the limitations found. We searched PubMed, and the grey literature to answer the question: Have studies involving patients diagnosed with cancer used the PPE to estimate cancer-specific survival? How do they justify the use of the PPE and what are the limitations pointed out? Out of 295 screened, 85 studies were included in this review. The two main characteristics of the PPE mentioned by the studies as justification were the fact that it is an unbiased estimator (83.5%) and that it produces comparable estimates among populations with different mortality rates from causes other than cancer (36.47%). No study pointed to a limitation due to the use of PPE. As a conclusion, the Pohar Perme Estimator is the gold standard for estimating net survival and should be more used in oncology, especially when dealing with population-based studies where the follow-up time is long, making high the probability of death from causes other than cancer.

## 1. Introduction

Cancer is a non-communicable chronic disease widely recognized as a global health problem for which effective solutions are lacking due to slow progress in the prevention and control. The Global Burden of Disease (GBD) study was concerned with describing the burden of 29 cancer groups in 195 countries based on data from 1990 to 2017, and with the aim of providing necessary data for cancer control planning [1]. Global problems associated with cancer include social and health care inequities related to cancer incidence and mortality. As a result, most of the expected cancer deaths worldwide will occur in low- and middle-income countries. Preventive measures and reliable survival estimates can contribute to better targeting and controlling cancer deaths worldwide in the coming years [2,3].

Survival analysis can be seen as a tool to monitor and control cancer, as it can help set improvement goals and report progress in disease management in several domains. In this context, overall survival (OS) is one of the most used measures, especially when data comes from a sample of patients. The overall survival estimates the probability of death from any cause, that is, it considers death as an event, regardless of its cause [4]. Thus, OS is influenced by the risk of death from causes other than the cancer under study, especially when the population includes a high proportion of the elderly [3]. A high overall survival may be due to fewer deaths from other causes or due to fewer deaths from cancer. This influence limits the comparability of estimates between different countries. Thus, the estimation of cancer-specific survival, that is, “the survival rate so far as the disease under study is concerned” [5], adds important information to assess the behavior of the disease.

Cancer-specific survival can be estimated using widely known methods such as the Kaplan–Meier estimator and the Cox model. However, to use these methods it is necessary to know, for each patient, whether the death was from cancer or from another cause. Thus, the concept of relative survival (RS) was introduced with the aim of estimating cancer-specific survival without the need to know whether the observed death was due to cancer. RS is particularly useful when survival data comes from population cancer registries, where the cause of death may exist but may not be reliable. According to Ederer [6], the relative survival allows estimating the proportion of cancer deaths controlling for the difference in mortality from causes other than cancer [3]. Its main assumption is to consider that the expected mortality of the general population correctly reflects mortality from other causes (besides cancer) [7], that is, that cancer deaths are an insignificant proportion of all deaths [8]. However, this assumption is debatable, especially in studies of more common cancers or at more advanced ages, making the concept of net survival (NS) more adequate in these cases [5]. Net survival assumes that the disease under study is the only possible cause of death and is estimated by decomposing the observed hazard into the hazard to the disease and that due to other causes. In summary, net survival and relative survival are different ways of estimating specific survival.

In this context, in 2012, Pohar Perme proposed a non-parametric estimator (PPE) for net survival that does not require modeling such as the other methods that were being used until then [5]. The PPE assumes that the time from death to the disease and the time from death to other causes are conditionally independent, given a known set of covariates. In this case, it is considered that the risk due to other causes is caused by the mortality of the population, and, thus, the observed risk is greater than the risk of the population, thus leading to the idea of an unbiased estimator of net survival [5,9,10].

Despite the complexity in the definitions of survival analysis methods, PPE is the method suggested in the literature for estimating disease-specific survival when the cause of death is unknown [5]. Large-scale multinational survival studies such as SUDCAN e CONCORD have used PPE. Given the absence of a review of this topic in oncology, this scoping review was performed with the objective of mapping oncological studies that used the PPE to know which context they are being used and what the authors point out as justification for its use and the limitations found.

## 2. Methods

This study followed the proposal of a scoping review as recommended by the Joana Briggs Institute (JBI) [11]. We chose to use scoping review because of its usefulness for summarizing, disseminating, and mapping the fundamental concepts of a given methodology [12]. To construct the research question, we used the strategy population, concept, and context: P = patients diagnosed with cancer, C = net survival, and C = cancer survival. Based on these definitions, this review intends to answer the following question: Have studies involving patients diagnosed with cancer used the PPE to estimate cancer-specific survival? How do they justify the use of the PPE and what are the limitations pointed out?

### 2.1. Search Strategy

The search was carried out in PubMed and grey literature such as Google Scholar, dissertations and theses published in the CAPES repository and hand search of the references of the studies resulting from the primary electronic search. It was guided by the Medical Subject Headings (MeSH) descriptors “net survival” and “cancer survival”. We felt that these two descriptors would capture most of the published literature to answer our research question. Considering that the PPE was proposed in 2012, the search was limited to the period from 1 January 2012 to 23 February 2022. No filters were applied to the language or status of the publication.

To identify the studies included in this review, the Boolean operators “OR” and “AND” were used to compose the search alternatives between each group of words and to unite the different groups, respectively. The PubMed search terms were the following: (cancer survival) AND (“net survival”); filters: from 2012 to 2022. The MeSH search terms were the following: (“cancers” OR “cancerated” OR “canceration OR “cancerization” OR “cancerized” OR “cancerous” OR “neoplasms” OR “neoplasms” OR “cancer” OR “cancers”) AND (“mortality” OR “mortality” OR “survival”).

### 2.2. Selection Process

Searches obtained from electronic databases were merged using Zotero reference manager. In the next stage, duplicate records were removed, and the studies selection was carried out in two stages by two independent reviewers and potential disagreements were resolved by a third reviewer. In the first stage, the titles and abstracts of the identified references were evaluated according to the inclusion criteria. In the second stage, the full-text evaluations of the selected studies confirm or not the inclusion in this review.

### 2.3. Data Collection

Data were extracted using an Excel spreadsheet created by the authors exclusively for this study containing characteristics of the study (year of publication, country, type of cancer, and objectives) and specific information related to this study objective (factors used to justify the use of PPE, statistical software used to perform PPE, and limitations reported by the authors of the included studies). Data extraction from three studies was performed as a pilot test to guarantee the quality of the data collection.

### 2.4. Analysis of the Evidence and Presentation of the Results

The extracted data were summarized using absolute and relative frequencies and were presented in a discursive manner throughout the results section.

## 3. Results

**Figure 1:**
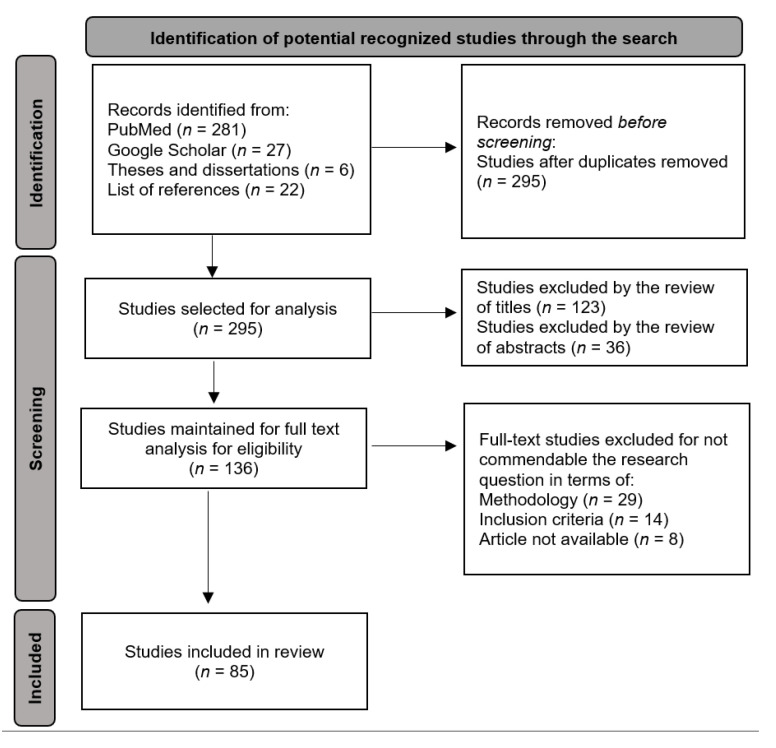
PRISMA flowchart for study selection and review.

The results of the search, the selection process, and the reasons for exclusion are presented in the study flow diagram (Figure 1). Some studies addressed other measures to estimate net survival in addition to PPE. Following the objectives of this review, we will present results only in relation to the use of PPE. Overall, 85 studies were included in this scoping review.

Most of the included studies were published between 2015 and 2018, many (*n* = 52; 61%) involve patients from different parts of the world and various types of cancer (*n* = 26; 31%). The vast majority (*n* = 24; 28%) estimated the net survival at 5 years (Table 1). Detailed information is provided in the Supplementary Materials (Table S1).

All included studies used data from population-based cancer registries and many of them (*n* = 29; 34%) were global in scope to provide net survival estimates, trends, and international variations in survival for various types of cancer. Among these studies are world reference studies such as SURVMARK-2, CONCORD, and SUDCAN (Table S1). Among studies that investigated a single type of cancer individually, breast, melanoma, ovarian, and esophageal cancers were the most frequently analyses (*n* = 5; 6%), followed by lung, colon, liver, and prostate cancer (*n* = 4; 5%).

The studies included were unanimous in pointing out that PPE is indicated for long follow-ups when compared to other estimation methods, because the justification converged in the fact that the longer the follow-up time, the longer the information censorship mechanism, leading to biases in the estimation if other methods were used [9,13,14] In fact, only six (7.1%) of the included estimated net survivals in less than 5 years elapsed since the diagnosis of the cancer.

About statistical analysis, overall, 83 (97.6%) of the included studies included in this review provided age-standardized estimates using the International Cancer Survival Standard (ICSS) weights, and a large part was categorized into five age groups: 15–44, 45–54, 55–64, 65–74, and 75–99 years [15]. Pohar Perme’s approach to estimating net survival takes population differences into account, and for that, the background mortality of the general population is derived from life tables of each cancer registry containing all-cause mortality rates by sex, age, and calendar year for the period analyzed in the respective jurisdiction [7,8,16]. The variables most frequently observed in net survival estimates were age, period/year of diagnosis, tumor stage, location/type of cancer, and country. Only 21% of the studies assessed race/ethnicity.

To answer our research question, we categorized the justifications for the use of PPE and the limitations pointed out by the authors. The summary of these results is presented in Table 2 together with the result of the software used for the analysis of each study included.

### 3.1. Justification for Using the PPE

Most studies (*n* = 71; 83.53%) justified the use of the PPE because it is one of the only two unbiased estimators for cancer-specific survival.

The second most cited justification (*n* = 31; 36.47%) is about the PPE allowing the resulting survival estimates to be compared across countries, regions, or different periods of time. According to its definition, the use of PPE considers, when calculating its estimates, the risk of death of the population without cancer, thus allowing this comparability.

Another argument cited by 19 (22.35%) studies in this review is the fact that PPE has been recognized as an important epidemiological indicator. This is because it estimates specific cancer survival through the concept of excess mortality, that is, it eliminates influences from causes of death other than cancer.

### 3.2. Limitations Reported in the Included Studies

Several limitations were mentioned by the authors of the studies included in this review and are summarized below to try to understand the difficulties encountered in applying the PPE. To find out which studies reported which limitations, see Table S1 of the Supplementary Material.

#### 3.2.1. Follow-Up Time and Vital Status

A total of 10 (11.76%) studies cited limitations in this category. Difficulties in detecting cases in early screening, a lack of data on vital status, a short follow-up time, and a reduced number of patients at risk for long follow-up periods were cited.

#### 3.2.2. Comparability and Classification Bias

One of the potentialities of the PPE highlighted in the original paper is that it allows survival estimates from different countries, regions, or time periods to be comparable. Despite confirming this potential, 41 (48.20%) studies in this review pointed out as a limitation the impossibility of comparing their results with others from other countries. However, none of the justifications were related to the use of PPE, but rather to differences such as diagnostic techniques, tumor classifications, and life tables not stratified by socio-economic variables, among others. Another limitation cited as a justification to explain possible differences in cancer survival estimates across countries is they may be due to inequalities in population cancer registry practices, including differences in tumor categorizations, determinations of prognostic factors, errors in death records, and inadequate or incomplete life tables. 

#### 3.2.3. Missing Data

A potential factor that can lead to problems in the interpretation of the results produced by the PPE is the lack of data. In cancer studies, some prognostic factors are very important for a comprehensive understanding related to survival. In line with this explanation, 29 (34.11%) studies in this review cited the lack of information regarding prognostic factors as a limitation of their work. Information on some variables were cited as scarce: staging at diagnosis, type of treatment, histology, anatomical site, and cancer morphology. 

#### 3.2.4. Sample Size

To apply the PPE, it is necessary to estimate overall survival by group of patients depending on factors such as age, sex, and race, among others. Therefore, when the absolute number of cases is small, which is common for young patients, the estimates produced by the PPE should be interpreted with caution. Authors of 17 (20.00%) studies in this review emphasized difficulties in obtaining age-standardized estimates due to the small sample size in certain groups of patients (sex and race).

### 3.3. Software

While most studies calculated the Pohar-Perme estimator using the stns command implemented in Stata (*n* = 31; 36.5%), or the implementation method obtained in the SEER*Stat program (*n* = 19; 22.3%), only 11 studies (12.94%) performed the analysis in the R program using the relsurv package as indicated by the author of the Pohar Perme Estimator. It is also worth mentioning that one study (1.18%) performed the analysis using the SAS program.

## 4. Discussion

Overall survival is the most used measure to understand the prognosis of cancer patients. By considering death from any cause as the event of interest is therefore influenced by the risk of death from causes other than cancer. In this sense, estimating cancer-specific survival adds important information to access the behaviour of the disease. The PPE estimates cancer-specific survival using the concept of net survival. Whether the specific cause is cancer may be interpreted as cancer survival in the hypothetical situation in which cancer of interest is the only possible cause of death [5]. To use the PPE, it is not necessary to know the cause of death for each patient in the sample. In addition, it has been suggested for comparisons of survival of different populations and is considered an important epidemiological indicator. In this scoping review, we found 85 studies published between 2012 and 2022 that used the PPE estimator to obtain net survival estimates for cancer patients. The small number of studies found showed the paucity of research using the PPE in the field of oncology. This may be since there are few population-based studies in the field of cancer or the lack of dissemination of this methodology.

This review sought to understand the potential of the PPE by summarizing what authors who have already used this estimator bring as a justification for using it. Most authors of the studies in this review cited the fact that the PPE is an unbiased estimator. In fact, being unbiased is the main characteristic of the PPE since the magnitude of the errors imposed by the classical cancer specific-survival estimator through the relative survival approach, namely, EDERER I, Ederer II and Hakulinen, can be very important. In addition, the fact that the PPE is the only non-biased estimator makes it the preferred method for estimating net survival [13].

Another important feature of the PPE cited by several studies in this review is its suitability for producing estimates that can be compared between different populations. The comparability power of the PPE meets the great interest of oncologists to assess their efforts to improve cancer outcomes. The PPE assumes that the risk of death from other causes is completely specified by overall mortality. Thus, it is built on the assumption of excess risk, which allows for comparability between populations with different risks of mortality from other causes [5].

Our review also shows that the limitations highlighted by studies in the field of oncology that used the PPE are not limitations imposed by the estimator, but inherent limitations of the use of data from population records and specificities of the oncology area, such as the different ways of grouping types of cancer or staging at diagnosis.

Finally, we must say that our study should be interpreted in the context of a scoping review methodology that does not assess the quality of the included studies.

## 5. Conclusions

This review was able to demonstrate the usefulness of net survival in cancer studies when estimated using PPE. No limitations regarding the use of PPE were pointed out by the studies included in this scoping review and the justifications pointed out by the authors are in line with studies that theoretically compare PPE with other estimators for specific cancer survival. Thus, we suggest the Pohar-Perme estimator to estimate net survival in oncology studies that are based on data from population records. We also emphasize that the use of the Pohar-Perme estimator is easy to implement and that the estimates should be standardized by age when the objective is to compare them with other populations, since age is an important prognostic factor. We hope that the PPE will be more used in studies in the oncology area, as it is an adequate epidemiological indicator to help establish control strategies and improve the survival of these patients.

## Figures and Tables

**Table 1:** Summary of the characteristics of the included studies.

Features of the Reviewed Studies	*n*	%
PUBLICATION YEAR		
2010–2014	4	4.71
2015–2018	52	61.18
2019–2022	29	34.12
COUNTRIES		
USA	12	14.12
France	12	14.12
Other European countries	12	14.12
Other countries	20	23.53
Worldwide	29	34.12
TYPES OF CANCER		
Breast	5	5.88
Lung	4	4.71
Ovary	5	5.88
Colon	4	4.71
Prostate	4	4.71
Stomach/esophagus	5	5.88
Melanoma	5	5.88
Liver	4	4.71
Several cancers *	26	30.59
Others	23	27.06
FOLLOW-UP		
1 and 3 years	6	7.1
1 and 5 years	20	23.5
1, 3, and 5 years	14	16.5
5 years	24	28.2
5 and 10 years	7	8.2
5, 10, and 15 years	5	5.9
More than 15 years of follow-up	2	2.4
Other combinations of segments	7	8.2

* Studies that evaluated more than one type of cancer were classified as having several cancers.

**Table 2:** Distribution of included studies according to PPE use justification and limitations.

Results from Included Studies Header	*n* **	% **
Justification for using the PPE		
1. Unbiased estimator	71	83.53
2. Important epidemiological indicator	19	22.35
3. Comparability of estimates	31	36.47
4. Not available	14	16.47
Limitations reported in the included studies		
1. Follow-up time and vital status	10	11.76
2. Comparability and classification bias	41	48.20
3. Missing data	29	34.11
4. Sample size	17	20.00
5. Others	1	1.18
6. Not available	18	21.18
Software used to calculate PPE		
STATA	31	36.47
R	11	12.94
SEER * Stat	19	22.35
SAS	1	1.18
Not available	23	27.06

** Studies may appear in more than one category.

## Data Availability

The data presented in this study are available on request from the corresponding author.

## Supplementary Materials

The following supporting information can be downloaded at: https://www.mdpi.com/article/10.3390/cancers14143304/s1, Table S1: Description of studies included in the scoping review according to title, Authorship/Year of publication, Country, Follow-up, Objective, Justifications and Limitations.

## References

[1] Fitzmaurice C., Akinyemiju T.F., Al Lami F.H., Alam T., Alizadeh-Navaei R., Allen C., Alsharif U., Alvis-Guzman N., Amini E., Global Burden of Disease Cancer Collaboration (2018). Global, Regional, and National Cancer Incidence, Mortality, Years of Life Lost, Years Lived With Disability, and Disability-Adjusted Life-Years for 29 Cancer Groups, 1990 to 2016: A Systematic Analysis for the Global Burden of Disease Study. JAMA Oncol.

[2] Influência das Iniquidades Sobre Mortes por Câncer é tema da Revista Brasileira de Cancerologia. https://www.inca.gov.br/noticias/influencia-das-iniquidades-sobre-mortes-por-cancer-e-tema-da-revista-brasileira-de.

[3] Pohar Perme M., Estève J., Rachet B. (2016). Analysing Population-Based Cancer Survival – Settling the Controversies. BMC Cancer.

[4] Mariotto A.B., Noone A.-M., Howlader N., Cho H., Keel G.E., Garshell J., Woloshin S., Schwartz L.M. (2014). Cancer Survival: An Overview of Measures, Uses, and Interpretation. JNCI Monogr..

[5] Perme M.P., Stare J., Estève J. (2012). On Estimation in Relative Survival. Biometrics.

[6] Ederer F., Axtell L.M., Cutler S.J. (1961). The Relative Survival Rate: A Statistical Methodology. Natl. Cancer Inst. Monogr..

[7] Coviello E., Seppä K., Dickman P.W., Pokhrel A. (2015). Estimating Net Survival Using a Life-Table Approach. Stata J..

[8] Cho H., Howlader N., Mariotto A.B., Cronin K.A. (2011). Estimating Relative Survival for Cancer Patients from the SEER Program Using Expected Rates Based on Ederer I versus Ederer II Method. https://surveillance.cancer.gov/reports/tech2011.01.pdf.

[9] Seppä K., Hakulinen T., Pokhrel A. (2015). Choosing the Net Survival Method for Cancer Survival Estimation. Eur. J. Cancer.

[10] Hakulinen T., Seppä K., Lambert P.C. (2011). Choosing the Relative Survival Method for Cancer Survival Estimation. Eur. J. Cancer.

[11] About-Jbi - About JBI|Joanna Briggs Institute. https://jbi.global/about-jbi.

[12] Aromataris E., Munn Z. (2020). JBI Manual for Evidence Synthesis.

[13] Roche L., Danieli C., Belot A., Grosclaude P., Bouvier A.-M., Velten M., Iwaz J., Remontet L., Bossard N. (2013). Cancer Net Survival on Registry Data: Use of the New Unbiased Pohar-Perme Estimator and Magnitude of the Bias with the Classical Methods. Int. J. Cancer.

[14] Danieli C., Remontet L., Bossard N., Roche L., Belot A. (2012). Estimating Net Survival: The Importance of Allowing for Informative Censoring. Stat. Med..

[15] Corazziari I., Quinn M., Capocaccia R. (2004). Standard Cancer Patient Population for Age Standardising Survival Ratios. Eur. J. Cancer.

[16] Baili P., Micheli A., De Angelis R., Weir H.K., Francisci S., Santaquilani M., Hakulinen T., Quaresma M., Coleman M.P., The CONCORD Working Group (2008). Life Tables for World-Wide Comparison of Relative Survival for Cancer (CONCORD Study). Tumori J..

