# Supplementary material for: Net Survival in Survival Analyses for Patients with Cancer: A Scoping Review
Source: Cancers (Basel). 2022 Jul 6;14(14):3304. doi: 10.3390/cancers14143304 (PMC9322882; doi:10.3390/cancers14143304)
Supplement: Supplementary file 1 [file cancers-14-03304-s001.zip › cancers-1700408-supplementary.pdf]

Supplementary Material: Net Survival in Survival Analyses for Patients with Cancer: A Scoping Review

Camila Macedo Lima Nagamine, Bárbara Niegia Garcia de Goulart and Patrícia Klarmann Ziegelmann

Table S1. Description of studies included in the scoping review according to title, Authorship/Year of publication, Country, Follow-up, Objective, Justifications and Limitations.

Author/Year*	Country	Cancer	Follow-Up	Objective	Justifications**	Limitations***
Monnereau [1] 2012	France	hematol.	5 and 10	Estimate the net survival of 5 and 10 years of patients with MH by gender, age classes and ten main subtypes of MH.	1 and 2	6
Jooste [2] 2013	France	various cancers	5 and 10	Highlight the changes in net survival over the reporting periods and according to age in relation to 33 cancer sites.	1 and 2	6
Roche [3] 2013	France	various cancers	5, 10 and 15	To investigate the magnitude of the errors made by four methods of relative survival (Ederer I, Hakulinen, Ederer II and a univariate regression model) vs. PPE as a reference and examined the influence of follow-up time, cancer prognosis and age on errors made.	1	1 and 5
Rollot [4] 2013	France	colorectal	5 and 10	Analyze long-term net survival trends for colorectal cancer using a population-based digestive cancer registry.	1, 2 and 3	6
Allemani [5] 2015	World Study	various cancers	5	Analyze progress towards the goal of reductions in premature cancer deaths and improvements in quality of life and cancer survival.	1 and 2	1, 3 and 4
Colonna [6] 2015	France	thyroid	5 and 10	Describe changes in the incidence of thyroid papilar cancer and its geographic variations and provide estimates of net survival.	1	6
Morris [7] 2015	England	breast	5	Describe the differences in survival between ethnic groups and deprivation, and whether screening status has an impact on them.	1	1, 2 and 4
Schaffar [8] 2015	Geneva	breast	1 and 20	Compare the two data configurations, cause-specific and relative survival, when estimating long-term net survival.	1 and 2	2
Seppä [9] 2015	Finland	various cancers	5, 10 and 14	Investigate how much the results obtained by the two methods (EdererII and PP) differ and under what conditions the new method can be recommended in practice.	1	1
Seppä [10] 2015	Finland	various cancers	5, 10 and 15	Compare the non-standard estimators of Ederer II, Age-standardized Ederer II and Liquid Survival Pohar Perme through a simulation, which reflects the actual behavior of cancer incidence and survival over age and civilian time at different cancer sites.	1	2
Trétarre [11]	France	ovary	1, 5 and 10	Describe the incidence, mortality and survival of ovarian cancer in France	1	6

2015						
Bouvier [12] 2016	France	straight	5 and 10	Report trends in the incidence and survival of anal cancer in France prior to the implementation of the human papilloma virus vaccine	1, 2 and 3	3
Glória [13] 2017	World Study	stomach	1 and 5	Compare the net survival of stomach cancer and provide trends in net survival and the dynamics of excessive mortality rates.	1,2 and 3	3
Lenartova [14] 2016	Norway	leukemia	5, 10 and 15	Analyze trends in the incidence and survival of chronic lymphocytic leukemia in Norway.	1 and 2	1 and 3
Perme [15] 2016	Slovenia	various cancers	10	Make a clear distinction between measures and describe their interpretation	1 and 2	6
Allemani [16] 2017	USA	various cancers	5	Analyze the differences in survival by race and stage in diagnosis.	1 and 3	
Antunes [17] 2017	World Study	uterus	1 and 5	Estimate the differences between countries in standardized net survival, assess time trends and excessive mortality rates.	1, 2 and 3	2 and 3
Antunes [18] 2017	Portugal	various cancers	1 and 5	Evaluate the liquid survival of cancer and analyze the trends of excess mortality for the types of malignant tumors.	1, 2 and 3	3 and 2
Benard [19] 2017	USA	cervical	1, 3 and 5	With US data from CONCORD-2, the objective was to analyze survival by race, stage and geographic area.	1	2 and 3
Bonaventure [20] 2017	World Study	childhood leukemia	5	Examine global trends in the survival of acute lymphoblastic leukemia of precursor cells in children, along with trends in the survival of acute myeloid leukemia and other types of childhood leukemia.	1	2 and 4
Bordoni [21] 2017	World Study	lung	1 and 5	Compare trends in net lung cancer survival and trends in excessive mortality rates.	1 and 3	2
Bouvier [22] 2017	World Study	pancreas	1 and 5	Compare the net survival of pancreatic cancer and provide trends in the liquid survival and dynamics of excessive mortality rates after diagnosis.	1, 2 and 3	3 and 4
Chirlaque [23] 2017	World Study	ovary	1 and 5	Compare trends in net ovarian cancer survival and trends in excessive mortality rates	1 and 2	4
Cowppli-Bony [24] 2017	France	various cancers	5, 10 and 15	Provide updates to estimates of net survival in 5 and 10 years, survival trends at 15 years after cancer diagnosis.	4	2
Crocetti [25] 2017	world	breast	1 and 5	Compare trends in net breast cancer survival and trends in excessive mortality rates	1, 2 and 3	1
Daubisse-Marliac [26] 2017	France	penile	1, 3 and 5	Determine whether there has been an evolution in the incidence and survival of penile cancer over time in France.	4	6
Delacour-Billon [27] 2017	France	breast	5 and 10	Estimate the short- and long-term survival of interval breast cancers and compare them to clinically detected cancers, taking into account prognostic characteristics	1 and 2	3

Deloumeaux [28] 2017	Guadeloupe	prostate	1, 3 and 5	Estimate the short- and long-term survival of interval breast cancers and compare them to clinically detected cancers, taking into account prognostic characteristics	1	2 and 3
Faivre [29] 2017	World Study	colon	1 and 5	Compare the net survival of colon cancer and provide trends in the liquid survival and dynamics of excessive mortality rates.	1, 2, and 3	6
Ferreti [30] 2017	World Study	liver	1 and 5	Compare the net survival of liver cancer and provide trends in net survival and dynamics of excessive mortality rates up to 5 years after diagnosis.	1 and 3	2
Galceran [31] 2017	World Study	melanoma	1 and 5	Compare the net survival of malignant melanoma cancer and provide trends in liquid survival and dynamics of excessive mortality rates up to 5 years after diagnosis.	1, 2 and 3	6
Grosclaude [32] 2017	World Study	prostate	1 and 5	Compare the net survival of prostate cancer and provide trends in net survival and the dynamics of excessive mortality rates.	1, 2, and 3	1 and 2
Guizard [33] 2017	World Study	various cancers	1 and 5	Compare the net survival of head and neck cancer and provide trends in net survival and the dynamics of excessive mortality rates.	1, 2, and 3	2
Haelens [34] 2017	World Study	cervical	1 and 5	Compare the net survival of cervical cancer and provide trends in net survival and the dynamics of excessive mortality rates.	1, 2, and 3	1
Jim [35] 2017	USA	stomach	1, 3 and 5	Use CONCORD-2 data to characterize differences in stomach cancer survival among whites and blacks in the United States by state and disease stage at diagnosis	1	2
Joseph [36] 2017	USA	rectal	1, 3 and 5	Use CONCORD-2 data to provide the first assessment of rectal cancer survival by stage in diagnosis and by race as a means of evaluating the efficacy of screening, diagnostic services and treatment for rectal cancer.	1, 2, and 3	2
Launoy [37] 2017	World Study	esophageal	1 and 5	Compare the net survival of esophageal cancer and provide trends in net survival and the dynamics of excessive mortality rates.	1, 2, and 3	3
Lepage [38] 2017	World Study	rectal	1 and 5	Compare the net survival of rectal cancer and provide trends in net survival and the dynamics of excessive mortality rates.	1, 2 and 3	3
Mangone [39] 2017	World Study	kidney	1 and 5	Compare the net survival of kidney cancer and provide trends in net survival and the dynamics of excessive mortality rates.	1, 2 and 3	3
Matz [40] 2017	World Study	ovary	5	Explore the international variation in survival of each group to help interpret international differences in the survival of all combined ovarian cancers. Examine the differences in specific stage survival.	1	2 and 3
Momim [41] 2017	USA	liver	1, 3 and 5	Use the CONCORD-2 study to report survival to liver cancer by race and stage in diagnosis.	1	2, 3 and 4
Richards [42] 2017	USA	lung	1, 3 and 5	Use the CONCORD-2 study to report survival to lung cancer by race and stage in diagnosis.	1	6
Schaffar [43] 2017	Geneva	various cancers	20	Comparison of net survival estimates for four cancer locations using cause-specific survival configuration and relative survival (PP) scenario.	1 and 2	6

Steele [44] 2017	USA	prostate	1, 3 and 5	Use CONCORD-2 data to examine cross-trends in prostate cancer survival up to 5 years among black and white men per stage of cancer.	1	1 and 2
Suteu [45] 2017	Romania	various cancers	5	Determine the trends in incidence of cutaneous melanoma and squamous cell carcinoma in Cluj County from 1998 to 2011 and net survival of 5 years.	1	4
Tai [46] 2017	USA	leukemia	1, 3 and 5	Describe the survival of children with Acute Leukemia in the United States, using the most comprehensive and up-to-date cancer registration data available by race and age.	1	2 and 3
van der Linden [47] 2017	Netherlands	vulvar glandular	5	To describe the incidence and survival of women with vulvar glandular neoplasms.	1 and 2	3 and 4
White [48] 2017	USA	colon	1, 3 and 5	Provide the first detailed assessment of colon cancer survival in the United States by state and stage in diagnosis and by race.	1	2
Afshar [49] 2018	Australia.	various cancers	5	Provide the first detailed assessment of colon cancer survival in the United States by state and stage in diagnosis and by race.	4	1 and 2
5Allemani [50] 2018	World Study	various cancers	5	Update global surveillance of cancer survival trends to include patients diagnosed by 2014	1	2 and 4
Baeyens-Fernández [51] 2018	Spain	breast	1, 3 and 5	Present a comprehensive population-based analysis of breast cancer epidemiology, including all indicators and age groups.	1	1 and 3
Bailey [52] 2018	USA	leukemia	5	Determine leukemia survival estimates in the United States from 1995 to 2009 according to subtype, gender, geographic area, and race.	1	2
Bellier [53] 2018	France	prostate	8	Compare incidence trends and prostate cancer survival rates among men aged ≥75 and 60-74 years.	1	3
Bravo [54] 2018	Colombia	various cancers	5	Disseminate the most recent cancer statistics in Cali for age-standardized incidence and mortality rates for all cancers and net survival estimates.	1	2
Ellison [55] 2018	Canada	various cancers	1, 3 and 5	Reveal progress in cancer outcomes in Canada since the early 1990s and on cancers for which there has apparently been no improvement in five-year net survival over a 20-year period.	1	6
Monteiro [56] 2018	Portugal	various cancers	5	Analyze the patterns of liquid survival of lip, oral cavity and oropharynx cancer using a population-based data set of cancer patients diagnosed in northern Portugal during the period 2000-2009.	1	2 and 3
Arnold [57] 2019	World Study	various cancers	1 and 5	Describe current patterns and recent trends in survival, incidence and mortality, to generate hypotheses with regard to the engines of these trends.	1 and 2	1 and 2
Alawadhi [58] 2019	Kuwait	various cancers	1,3 and 5	Evaluate the distribution of the stage at diagnosis in Kuwait and estimate the specific liquid stage survival at 1 and 5 years since diagnosis.	1	2, 3 and 4
Inoue [59] 2019	Japan	gynecological	1 and 5	Analyze survival among elderly patients with gynecological cancer		2

Afshar [60] 2020	Australia.	various cancers	5	Assess inequalities in cancer survival by distance from residence in Victoria, Australia.	4	2 and 3
Aoe [61] 2020	Japan	bladder	5	Examine long-term trends in sex difference in net bladder cancer survival using large-scale population-based cancer registry data from Osaka, Japan.	4	2, 3 and 4
Araghi [62] 2020	World Study	various cancers	1 and 5	Provide the most recent estimates of survival to colon and rectum cancer in seven high-income countries by age and stage at the time of diagnosis.	1	2
Botta [63] 2020	World Study	various cancers	5	Provide a detailed comparison of incidence and survival for rare cancers in the US and Europe.	1 and 2	2
Cabasag [64] 2020	World Study	ovary	1 and 3	Evaluate the differences in ovarian cancer survival by age and stage in diagnosis within and between seven high-income countries.	4	2 and 4
Di Carlo [65] 2020	USA	cutaneous melanoma	1	Describe the characteristics of patients diagnosed with melanoma and evaluate short-term survival trends for distant-stage disease.	1	6
Forjaz [66] 2020	Portugal	various cancers	5	Evaluate the evolution of cancer in a specific region of Portugal, through the three main metrics in cancer surveillance: incidence, survival and mortality.	4	4
Korovin [67] 2020	Ukraine	myeloma	5	Describe the burden of Myeloma Malígno in Ukraine in terms of incidence, mortality and survival by sex, age and stage for the period 2002-2013 and compare with European countries.	4	3
Morgan [68] 2020	World Study	esophageal	1 and 3	Report trends in the incidence and survival of esophageal cancer by histological subtype for seven countries with more than 20 years of data	4	2 and 3
Myklebust [69] 2020	World Study	various cancers	1 and 5	Evaluate the impact of applying different rules to determine DOI in survival estimates up to 5 years after diagnosis for eight cancer sites diagnosed in 2010-2014.	4	2
Riano [70] 2020	Colombia	various cancers	5	Provide a summary of the descriptive epidemiology of primary tumors in the urban population of Cali, Colombia.	1	2
Rutherford [71] 2020	Connecticut	ovary	5	Estimate 5-year survival using the approaches described in the previous sections to externally standardize by age the estimates for comparability.	1	6
Alves [72] 2021	Cuiabá and Vargen Grande	colorectal	5	To evaluate the net survival in 5 years of colorectal cancer cases in the cities of Cuiabá and Várzea Grande in the period from 2000 to 2009 using PBCR data in order to understand more specifically the situation of the disease in the area of coverage.	1 and 2	3
Araghi [73] 2021	World Study	lung	1 and 3	Evaluate the most up-to-date lung cancer survival statistics by gender and stage in diagnosis using population data from seven countries.	1	2 and 3
Aragon [74] 2021	Colombia	head and neck	5	Describe survival and changes in time trends in incidence and mortality rates with data from the Cali Cancer Population Registry during 1962-2018.	4	1 and 2
Arnold [75] 2021	World Study	esophageal and gastric	1 and 3	Examine the impact of the disease stage on diagnosis and histological subtype on international survival disparities in esophageal and gastric cancer.	1 and 2	2

Chang-Chan [76] 2021	Spain	myeloma	1, 3 and 5	Describe here a complete analysis of the trends of incidence, mortality and survival of multiple myeloma over a period of 23 years in both Spanish PBCRs.	4	2 and 3
Courant [77] 2021	France	myeloma	3	Evaluate the incidence and net survival of all patients living in Gironde newly diagnosed with Multiple Myeloma.	1	6
Hameed [78] 2021	California	colon	1, 2, 3, 4 and 5	Examine these disparities in the net survival of stage-specific colon cancer, gender, race/ethnicity, and socioeconomic cancer in California for adults diagnosed between 2004 and 2011.	1	4
John [79] 2021	England	bladder	1	Describe treatments and assess disease stage and gender survival for all newly diagnosed non-metastatic MBC in England in 2016 and observe associations between comorbidities and treatments	4	2, 3 and 4
Matz [80] 2021	USA	cervical	5	Analyze cervical cancer survival trends by race, stage and diagnosis period.	1	6
Mazzucco [81] 2021	Italy	liver	5	Examine survival by level of patient care for all combined patients and by age.	1	2 and 3
Morgan [82] 2021	World Study	lung	1 and 3	Investigate the potential impact on lung cancer survival estimates after statistically assigning cases with unspecified histology to a specific histological group.	4	2
Rutherford [83] 2021	World Study	liver	1 and 3	Compare the survival of liver cancer in ICBP jurisdictions, while trying to ensure that estimates are comparable through a series of sensitivity analyses.	1	2 and 3
Zadnik [84] 2021	Slovenia	various cancers	5	Describe the survival of Slovenian cancer patients diagnosed in the last twenty years.	1	6
Shin [85] 2022	Korea	vulvar	5 and 10	Report 20-year trends in the incidence and survival of vulvar cancer in Korea.	1	6

*Only the first author was mentioned in the text; **Justification: 1. Unbiased estimator, 2. Important epidemiological indicator, 3. Comparability of estimates, 4. Not available; ***Limitations: 1. Follow-up time and vital status, 2. Comparability and classification bias, 3. Missing data, 4. Sample size, 5. Others, 6. Not available.

Reference

1. Monnereau, A.; Troussard, X.; Belot, A.; Guizard, A.-V.; Woronoff, A.-S.; Bara, S.; Lapôtre-Ledoux, B.; Iwaz, J.; Tretarre, B.; Maynadié, M.; et al. Unbiased estimates of long-term net survival of hematological malignancy patients detailed by major subtypes in France. *Int. J. Cancer* **2012**, *132*, 2378–2387, https://doi.org/10.1002/ijc.27889.
2. Jooste, V.; Grosclaude, P.; Remontet, L.; Launoy, G.; Baldi, I.; Molinie, F.; Arveux, P.; Bossard, N.; Bouvier, A.-M.; Colonna, M.; et al. Unbiased estimates of long-term net survival of solid cancers in France. *Int. J. Cancer* **2012**, *132*, 2370–2377, https://doi.org/10.1002/ijc.27857.
3. Roche, L.; Danieli, C.; Belot, A.; Grosclaude, P.; Bouvier, A.-M.; Velten, M.; Iwaz, J.; Remontet, L.; Bossard, N. Cancer net survival on registry data: Use of the new unbiased Pohar-Perme estimator and magnitude of the bias with the classical methods. *Int. J. Cancer* **2012**, *132*, 2359–2369, https://doi.org/10.1002/ijc.27830.
4. Rollot, F.; Chauvenet, M.; Roche, L.; Hamza, S.; Lepage, C.; Faivre, J.; Bouvier, A.M. Long-term Net Survival in Patients With Colorectal Cancer in France. *Dis. Colon Rectum* **2013**, *56*, 1118–1124, https://doi.org/10.1097/dcr.0b013e31829f3436.
5. Allemani, C.; Weir, H.K.; Carreira, H.; Harewood, R.; Spika, D.; Wang, X.-S.; Bannon, F.; Ahn, J.V.; Johnson, C.J.; Bonaventure, A.; et al. Global surveillance of cancer survival 1995–2009: analysis of individual data for 25 676 887 patients from 279 population-based registries in 67 countries (CONCORD-2). *Lancet* **2014**, *385*, 977–1010, https://doi.org/10.1016/s0140-6736(14)62038-9.
6. Colonna, M.; Uhry, Z.; Guizard, A.V.; Delafosse, P.; Schvartz, C.; Belot, A.; Grosclaude, P. Recent trends in incidence, geographical distribution, and survival of papillary thyroid cancer in France. *Cancer Epidemiol.* **2015**, *39*, 511–518, doi:10.1016/j.canep.2015.04.015.
7. Morris, M.; Woods, L.M.; Rogers, N.; O'Sullivan, E.; Kearins, O.; Rachet, B. Ethnicity, deprivation and screening: survival from breast cancer among screening-eligible women in the West Midlands diagnosed from 1989 to 2011. *Br. J. Cancer* **2015**, *113*, 548–555, https://doi.org/10.1038/bjc.2015.204.
8. Schaffar, R.; Rachet, B.; Belot, A.; Woods, L. Cause-specific or relative survival setting to estimate population-based net survival from cancer? An empirical evaluation using women diagnosed with breast cancer in Geneva between 1981 and 1991 and followed for 20 years after diagnosis. *Cancer Epidemiology* **2015**, *39*, 465–472, https://doi.org/10.1016/j.canep.2015.04.001.
9. Seppä, K.; Hakulinen, T.; Läärä, E.; Pitkäniemi, J. Comparing net survival estimators of cancer patients. *Stat. Med.* **2015**, *35*, 1866–1879, https://doi.org/10.1002/sim.6833.
10. Seppä, K.; Hakulinen, T.; Pokhrel, A. Choosing the net survival method for cancer survival estimation. *Eur. J. Cancer* **2015**, *51*, 1123–1129, https://doi.org/10.1016/j.ejca.2013.09.019.
11. Trétarre, B.; Molinié, F.; Woronoff, A.-S.; Bossard, N.; Bessaoud, F.; Marrer, E.; Grosclaude, P.; Guizard, A.-V.; Delafosse, P.; Bara, S.; et al. Ovarian cancer in France: Trends in incidence, mortality and survival, 1980–2012. *Gynecol. Oncol.* **2015**, *139*, 324–329, https://doi.org/10.1016/j.ygyno.2015.09.013.
12. Bouvier, A.-M.; Belot, A.; Manfredi, S.; Jooste, V.; Uhry, Z.; Faivre, J.; Duport, N.; Grabar, S. Trends of incidence and survival in squamous-cell carcinoma of the anal canal in France. *Eur. J. Cancer Prev.* **2016**, *25*, 182–187, https://doi.org/10.1097/cej.0000000000000163.
13. Glória, L.; Bossard, N.; Bouvier, A.-M.; Mayer-Da-Silva, A.; Faivre, J.; Miranda, A. Trends in net survival from stomach cancer in six European Latin countries: results from the SUDCAN population-based study. *Eur. J. Cancer Prev.* **2017**, *26*, S32–S39, https://doi.org/10.1097/cej.0000000000000309.
14. Lenartova, A.; Johannesen, T.B.; Tjønnfjord, G.E. National trends in incidence and survival of chronic lymphocytic leukemia in Norway for 1953-2012: a systematic analysis of population-based data. *Cancer Med.* **2016**, *5*, 3588–3595, https://doi.org/10.1002/cam4.849.
15. Perme, M.P.; Estève, J.; Rachet, B. Analysing population-based cancer survival – settling the controversies. *BMC Cancer* **2016**, *16*, 1–8, https://doi.org/10.1186/s12885-016-2967-9.
16. Allemani, C.; Harewood, R.; Johnson, C.J.; Carreira, H.; Spika, D.; Bonaventure, A.; Ward, K.; Weir, H.K.; Coleman, M.P. Population-based cancer survival in the United States: Data, quality control, and statistical methods. *Cancer* **2017**, *123*, 4982–4993, https://doi.org/10.1002/cncr.31025.
17. Antunes, L.; Santos, L.L.; Bento, M.J. Survival from cancer in the north region of Portugal: results from the first decade of the millennium. *Eur. J. Cancer Prev.* **2017**, *26*, S170–S175, https://doi.org/10.1097/cej.0000000000000378.
18. Antunes, L.; Roche, L.; Bento, M.J. Trends in net survival from corpus uteri cancer in six European Latin countries: results from the SUDCAN population-based study. *Eur. J. Cancer Prev.* **2017**, *26*, S100–S106, https://doi.org/10.1097/cej.0000000000000294.

19. Benard, V.B.; Watson, M.; Saraiya, M.; Harewood, R.; Townsend, J.S.; Stroup, A.M.; Weir, H.K.; Allemani, C. Cervical cancer survival in the United States by race and stage (2001-2009): Findings from the CONCORD-2 study. *Cancer* **2017**, *123*, 5119–5137, https://doi.org/10.1002/cncr.30906.
20. Bonaventure, A.; Harewood, R.; A Stiller, C.; Gatta, G.; Clavel, J.; Stefan, D.C.; Carreira, H.; Spika, D.; Marcos-Gragera, R.; Peris-Bonet, R.; et al. Worldwide comparison of survival from childhood leukaemia for 1995–2009, by subtype, age, and sex (CONCORD-2): a population-based study of individual data for 89 828 children from 198 registries in 53 countries. *Lancet Haematol.* **2017**, *4*, e202–e217, https://doi.org/10.1016/s2352-3026(17)30052-2.
21. Bordoni, A.; Uhry, Z.; Antunes, L. Trends in net survival lung cancer in six European Latin countries: results from the SUDCAN population-based study. *Eur. J. Cancer Prev.* **2017**, *26*, S70–S76, https://doi.org/10.1097/cej.0000000000000299.
22. Bouvier, A.-M.; Bossard, N.; Colonna, M.; Garcia-Velasco, A.; Carulla, M.; Manfredi, S. Trends in net survival from pancreatic cancer in six European Latin countries: results from the SUDCAN population-based study. *Eur. J. Cancer Prev.* **2017**, *26*, S63–S69, https://doi.org/10.1097/cej.0000000000000303.
23. Chirlaque, M.-D.; Uhry, Z.; Salmerón, D.; Sánchez-Zapata, M.-I.; Zannoni, G.F.; Navarro, C. Trends in net survival from ovarian cancer in six European Latin countries: results from the SUDCAN population-based study. *Eur. J. Cancer Prev.* **2017**, *26*, S107–S113, https://doi.org/10.1097/cej.0000000000000302.
24. Cowppli-Bony, A.; Uhry, Z.; Remontet, L.; Voirin, N.; Guizard, A.-V.; Trétarre, B.; Bouvier, A.-M.; Colonna, M.; Bossard, N.; Woronoff, A.-S.; et al. Survival of solid cancer patients in France, 1989–2013: a population-based study. *Eur. J. Cancer Prev.* **2017**, *26*, 461–468, https://doi.org/10.1097/cej.0000000000000372.
25. Crocetti, E.; Bossard, N.; Uhry, Z.; Roche, L.; Rossi, S.; Capocaccia, R.; Faivre, J. Trends in net survival from 15 cancers in six European Latin countries: the SUDCAN population-based study material. *Eur. J. Cancer Prev.* **2017**, *26*, S3–S8, https://doi.org/10.1097/cej.0000000000000300.
26. Daubisse-Marliac, L.; Colonna, M.; Trétarre, B.; Defossez, G.; Molinié, F.; Jéhannin-Ligier, K.; Marrer, E.; Grosclaude, P. Long-term trends in incidence and survival of penile cancer in France. *Cancer Epidemiology* **2017**, *50*, 125–131, https://doi.org/10.1016/j.canep.2017.08.014.
27. Delacour-Billon, S.; Mathieu-Wacquant, A.L.; Campone, M.; Auffret, N.; Amossé, S.; Allioux, C.; Cowppli-Bony, A.; Molinié, F. Short-term and long-term survival of interval breast cancers taking into account prognostic features. *Cancer Causes Control* **2016**, *28*, 69–76, https://doi.org/10.1007/s10552-016-0836-0.
28. 28. Deloumeaux, J.; Bhakkan, B.; Eyraud, R.; Braud, F.; M'Ebobisse, N.M.; Blanchet, P.; Brureau, L. Prostate cancer clinical presentation, incidence, mortality and survival in Guadeloupe over the period 2008–2013 from a population-based cancer registry. *Cancer Causes Control* **2017**, *28*, 1265–1273, https://doi.org/10.1007/s10552-017-0962-3.
29. Faivre, J.; Bossard, N.; Jooste, V. Trends in net survival from colon cancer in six European Latin countries: results from the SUDCAN population-based study. *Eur. J. Cancer Prev.* **2017**, *26*, S40–S47, https://doi.org/10.1097/cej.0000000000000293.
30. Ferretti, S.; Bossard, N.; Binder-Fouchard, F.; Faivre, J.; Bordoni, A.; Biavati, P.; Frassoldati, A. Trends in net survival from liver cancer in six European Latin countries: results from the SUDCAN population-based study. *Eur. J. Cancer Prev.* **2017**, *26*, S56–S62, https://doi.org/10.1097/cej.0000000000000298.
31. Galceran, J.; Uhry, Z.; Marcos-Gragera, R.; Borràs, J. Trends in net survival from skin malignant melanoma in six European Latin countries: results from the SUDCAN population-based study. *Eur. J. Cancer Prev.* **2017**, *26*, S77–S84, https://doi.org/10.1097/cej.0000000000000306.
32. Grosclaude, P.; Roche, L.; Fuentes-Raspall, R.; Larrañaga, N. Trends in net survival from prostate cancer in six European Latin countries: results from the SUDCAN population-based study. *Eur. J. Cancer Prev.* **2017**, *26*, S114–S120, https://doi.org/10.1097/cej.0000000000000304.
33. Guizard, A.-V.; Uhry, Z.; de Raucourt, D.; Mazzoleni, G.; Sánchez, M.-J.; Ligier, K. Trends in net survival from head and neck cancer in six European Latin countries: results from the SUDCAN population-based study. *Eur. J. Cancer Prev.* **2017**, *26*, S16–S23, https://doi.org/10.1097/cej.0000000000000296.
34. Haelens, A.; Roche, L.; Bastos, J.; Woronoff, A.-S.; Zorzi, M.; Francart, J. Trends in net survival from cervical cancer in six European Latin countries: results from the SUDCAN population-based study. *Eur. J. Cancer Prev.* **2017**, *26*, S92–S99, https://doi.org/10.1097/cej.0000000000000292.
35. Jim, M.A.; Pinheiro, P.S.; Carreira, H.; Espey, D.K.; Wiggins, C.L.; Weir, H.K. Stomach cancer survival in the United States by race and stage (2001-2009): Findings from the CONCORD-2 study. *Cancer* **2017**, *123*, 4994–5013, https://doi.org/10.1002/cncr.30881.

36. Joseph, D.A.; Johnson, C.J.; White, A.; Wu, M.; Coleman, M.P. Rectal cancer survival in the United States by race and stage, 2001 to 2009: Findings from the CONCORD-2 study. *Cancer* **2017**, *123*, 5037–5058, https://doi.org/10.1002/cncr.30882.
37. Launoy, G.; Bossard, N.; Castro, C.; Manfredi, S. Trends in net survival from esophageal cancer in six European Latin countries: results from the SUDCAN population-based study. *Eur. J. Cancer Prev.* **2017**, *26*, S24–S31, https://doi.org/10.1097/cej.0000000000000308.
38. Lepage, C.; Bossard, N.; Dejardin, O.; Carmona-Garcia, M.C.; Manfredi, S.; Faivre, J. Trends in net survival from rectal cancer in six European Latin countries: results from the SUDCAN population-based study. *Eur. J. Cancer Prev.* **2017**, *26*, S48–S55, https://doi.org/10.1097/cej.0000000000000305.
39. Mangone, L.; Bossard, N.; Marcos-Gragera, R.; Pezzarossi, A.; Roncaglia, F.; Rossi, P.G. Trends in net survival from kidney cancer in six European Latin countries: results from the SUDCAN population-based study. *Eur. J. Cancer Prev.* **2017**, *26*, S121–S127, https://doi.org/10.1097/cej.0000000000000297.
40. Matz, M.; Coleman, M.; Carreira, H.; Salmerón, D.; Chirlaque, M.D.; Allemani, C.; Bouzbid, S.; Hamdi-Chérif, M.; Zaidi, Z.; Bah, E.; et al. Worldwide comparison of ovarian cancer survival: Histological group and stage at diagnosis (CONCORD-2). *Gynecol. Oncol.* **2016**, *144*, 396–404, https://doi.org/10.1016/j.ygyno.2016.11.019.
41. Momin, B.R.; Pinheiro, P.S.; Carreira, H.; Li, C.; Weir, H.K. Liver cancer survival in the United States by race and stage (2001-2009): Findings from the CONCORD-2 study. *Cancer* **2017**, *123*, 5059–5078, https://doi.org/10.1002/cncr.30820.
42. Richards, T.B.; Henley, S.J.; Puckett, M.C.; Weir, H.K.; Huang, B.; Tucker, T.C.; Allemani, C. Lung cancer survival in the United States by race and stage (2001-2009): Findings from the CONCORD-2 study. *Cancer* **2017**, *123*, 5079–5099, https://doi.org/10.1002/cncr.31029.
43. Schaffar, R.; Rachet, B.; Belot, A.; Woods, L.M. Estimation of net survival for cancer patients: Relative survival setting more robust to some assumption violations than cause-specific setting, a sensitivity analysis on empirical data. *Eur. J. Cancer* **2016**, *72*, 78–83, https://doi.org/10.1016/j.ejca.2016.11.019.
44. Do, C.B.S.; Li, J.; Huang, B.; Weir, H.K. Prostate cancer survival in the United States by race and stage (2001-2009): Findings from the CONCORD-2 study. *Cancer* **2017**, *123*, 5160–5177, https://doi.org/10.1002/cncr.31026.
45. Şuteu, O.; Blaga, M.L.; Nicula, F.; Şuteu, P.; Coza, O.F.; Achimaş-Cadariu, P.; Coza, D. Incidence trends and survival of skin melanoma and squamous cell carcinoma in Cluj County, Romania. *Eur. J. Cancer Prev.* **2017**, *26*, S176–S182, https://doi.org/10.1097/cej.0000000000000382.
46. Tai, E.W.; Ward, K.C.; Bonaventure, A.; Siegel, D.; Coleman, M. Survival among children diagnosed with acute lymphoblastic leukemia in the United States, by race and age, 2001 to 2009: Findings from the CONCORD-2 study. *Cancer* **2017**, *123*, 5178–5189, https://doi.org/10.1002/cncr.30899.
47. van der Linden, M.; Schuurman, M.; Bulten, J.; van der Aa, M.; Massuger, L.; de Hullu, J. Incidence and survival of glandular vulvar malignancies in the Netherlands. *Gynecol. Oncol.* **2017**, *144*, 553–557, https://doi.org/10.1016/j.ygyno.2017.01.020.
48. White, A.; Joseph, D.; Rim, S.H.; Mph, C.J.J.; Coleman, M.; Allemani, C. Colon cancer survival in the United States by race and stage (2001-2009): Findings from the CONCORD-2 study. *Cancer* **2017**, *123*, 5014–5036, https://doi.org/10.1002/cncr.31076.
49. Afshar, N.; English, D.R.; Thursfield, V.; Mitchell, P.L.; Marvelde, L.T.; Farrugia, H.; Giles, G.G.; Milne, R.L. Differences in cancer survival by sex: a population-based study using cancer registry data. *Cancer Causes Control* **2018**, *29*, 1059–1069, https://doi.org/10.1007/s10552-018-1079-z.
50. Allemani, C.; Matsuda, T.; Di Carlo, V.; Harewood, R.; Matz, M.; Nikšić, M.; Bonaventure, A.; Valkov, M.; Johnson, C.J.; Estève, J.; et al. Global surveillance of trends in cancer survival 2000–14 (CONCORD-3): analysis of individual records for 37 513 025 patients diagnosed with one of 18 cancers from 322 population-based registries in 71 countries. *Lancet* **2018**, *391*, 1023–1075, https://doi.org/10.1016/s0140-6736(17)33326-3.
51. Baeyens-Fernández, J.A.; Molina-Portillo, E.; Pollán, M.; Rodríguez-Barranco, M.; Del Moral, R.; Arribas-Mir, L.; Ramírez, E.S.-C.; Sánchez, M.-J. Trends in incidence, mortality and survival in women with breast cancer from 1985 to 2012 in Granada, Spain: a population-based study. *BMC Cancer* **2018**, *18*, 1–14, https://doi.org/10.1186/s12885-018-4682-1.
52. Bailey, C.; Richardson, L.C.; Allemani, C.; Bonaventure, A.; Harewood, R.; Moore, A.R.; Stewart, S.L.; Weir, H.K.; Coleman, M.P.; CONCORD Working Group (US members) Adult leukemia survival trends in the United States by subtype: A population-based registry study of 370,994 patients diagnosed during 1995-2009. *Cancer* **2018**, *124*, 3856–3867, https://doi.org/10.1002/cncr.31674.
53. Bellier, A.; Colonna, M.; Delafosse, P.; Seigneurin, A. Incidence of prostate cancer and net survival by grade in a geriatric population: A population-based study in a French administrative entity from 1991 to 2013. *Cancer Epidemiology* **2018**, *56*, 60–66, https://doi.org/10.1016/j.canep.2018.07.007.

54. Bravo, L.E.; García, L.S.; Collazos, P.; Carrascal, E.; Ramirez, O.; Collazos, T.; Cortés, A.; Núñez, M.; Millan, E. Reliable information for cancer control in Cali, Colombia. *Colomb. Medica* **2018**, *49*, 23–34, https://doi.org/10.25100/cm.v49i1.3689.
55. Ellison, L.F. Progress in net cancer survival in Canada over 20 years.. *Public Heal. Rep. (1896-1970)* **2018**, *29*, 10–18.
56. Monteiro, L.S. Survival probabilities and trends for lip, oral cavity and oropharynx cancers in the Northern Region of Portugal in the period 2000–2009. *ecancermedicalscience* **2018**, *12*, 855, https://doi.org/10.3332/ecancer.2018.855.
57. Arnold, M.; Rutherford, M.; Bardot, A.; Ferlay, J.; Andersson, T.M.-L.; Myklebust, T. Åge; Tervonen, H.; Thursfield, V.; Ransom, D.; Shack, L.; et al. Progress in cancer survival, mortality, and incidence in seven high-income countries 1995–2014 (ICBP SURVMARK-2): A population-based study. *Lancet Oncol.* **2019**, *20*, 1493–1505, doi:10.1016/s1470-2045(19)30456-5.
58. Alawadhi, E.; Al-Awadi, A.; Elbasmi, A.; Coleman, M.; Allemani, C. Cancer Survival by Stage at Diagnosis in Kuwait: A Population-Based Study. *J. Oncol.* **2019**, *2019*, 1–9, https://doi.org/10.1155/2019/8463195.
59. Inoue, S.; Ito, H.; Hosono, S.; Hori, M.; Matsuda, T.; Mizuno, M.; Kato, K.; Matsuo, K. Net Survival of Elderly Patients with Gynecological Cancer Aged Over 75 Years in 2006-2008. *Asian Pac. J. Cancer Prev.* **2019**, *20*, 437–442, https://doi.org/10.31557/apjcp.2019.20.2.437.
60. Afshar, N.; English, D.R.; Blakely, T.; Thursfield, V.; Farrugia, H.; Giles, G.G.; Milne, R.L. Differences in cancer survival by area-level socio-economic disadvantage: A population-based study using cancer registry data. *PLoS ONE* **2020**, *15*, e0228551, https://doi.org/10.1371/journal.pone.0228551.
61. Aoe, J.; Ito, Y.; Fukui, K.; Nakayama, M.; Morishima, T.; Miyashiro, I.; Sobue, T.; Nakayama, T. Long-term trends in sex difference in bladder cancer survival 1975-2009: A population-based study in Osaka, Japan. *Cancer Med.* **2020**, *9*, 7330–7340, https://doi.org/10.1002/cam4.3382.
62. Araghi, M.; Arnold, M.; Rutherford, M.J.; Guren, M.G.; Cabasag, C.J.; Bardot, A.; Ferlay, J.; Tervonen, H.; Shack, L.; Woods, R.; et al. Colon and rectal cancer survival in seven high-income countries 2010–2014: variation by age and stage at diagnosis (the ICBP SURVMARK-2 project). *Gut* **2020**, *70*, 114–126, https://doi.org/10.1136/gutjnl-2020-320625.
63. Botta, L.; Gatta, G.; Trama, A.; Bernasconi, A.; Sharon, E.; Capocaccia, R.; Mariotto, A.B.; the RARECAREnet Working Group Incidence and survival of rare cancers in the US and Europe. *Cancer Med.* **2020**, *9*, 5632–5642, https://doi.org/10.1002/cam4.3137.
64. Cabasag, C.J.; Butler, J.; Arnold, M.; Rutherford, M.; Bardot, A.; Ferlay, J.; Morgan, E.; Møller, B.; Gavin, A.; Norell, C.H.; et al. Exploring variations in ovarian cancer survival by age and stage (ICBP SurvMark-2): A population-based study. *Gynecol. Oncol.* **2020**, *157*, 234–244, https://doi.org/10.1016/j.ygyno.2019.12.047.
65. Di Carlo, V.; Estève, J.; Johnson, C.; Girardi, F.; Weir, H.K.; Wilson, R.J.; Minicozzi, P.; Cress, R.D.; Lynch, C.F.; Pawlish, K.S.; et al. Trends in short-term survival from distant-stage cutaneous melanoma in the United States, 2001-2013 (CONCORD-3). *JNCI Cancer Spectr.* **2020**, *4*, pkaa078, https://doi.org/10.1093/jncics/pkaa078.
66. Forjaz, G.; Chen, H.-S.; Howlader, N.; Rego, R.; Rodrigues, V.; Mariotto, A.B. Measuring progress against cancer in the Azores, Portugal: Incidence, survival, and mortality trends and projections to 2025. *Cancer Epidemiology* **2020**, *69*, 101810, https://doi.org/10.1016/j.canep.2020.101810.
67. Korovin, S.; Fedorenko, Z.; Michailovich, Y.; Kukushkina, M.; Sekerija, M.; Ryzhov, A. Burden of malignant melanoma in Ukraine in 2002–2013: incidence, mortality and survival. *Exp. Oncol.* **2020**, *42*, 324–329, https://doi.org/10.32471/exp-oncology.2312-8852.vol-42-no-4.15334.
68. Morgan, E.; Soerjomataram, I.; Gavin, A.T.; Rutherford, M.J.; Gatenby, P.; Bardot, A.; Ferlay, J.; Bucher, O.; De, P.; Engholm, G.; et al. International trends in oesophageal cancer survival by histological subtype between 1995 and 2014. *Gut* **2020**, https://doi.org/10.1136/gutjnl-2020-321089.
69. Myklebust, T..; Andersson, T.; Bardot, A.; Vernon, S.; Gavin, A.; Fitzpatrick, D.; Jerm, M.B.; Rutherford, M.; Parkin, D.M.; Sasieni, P.; et al. Can different definitions of date of cancer incidence explain observed international variation in cancer survival? An ICBP SURVMARK-2 study. *Cancer Epidemiology* **2020**, *67*, 101759, https://doi.org/10.1016/j.canep.2020.101759.
70. Riano, I.; Bravo, P.; Bravo, L.E.; Garcia, L.S.; Collazos, P.; Carrascal, E. Incidence, Mortality, and Survival Trends of Primary CNS Tumors in Cali, Colombia, From 1962 to 2019. *JCO Glob. Oncol.* **2020**, *6*, 1712–1720, https://doi.org/10.1200/go.20.00368.
71. Rutherford, M.J.; Dickman, P.W.; Coviello, E.; Lambert, P.C. Estimation of age-standardized net survival, even when age-specific data are sparse. *Cancer Epidemiology* **2020**, *67*, 101745, https://doi.org/10.1016/j.canep.2020.101745.

72. Alves, C.M.M.; Prado, P.C.d.O.; Bastos, R.R. Net survival for colorectal cancer in Cuiabá and Várzea Grande (state of Mato Grosso), Brazil. *ecancermedicalscience* **2021**, *15*, https://doi.org/10.3332/ecancer.2021.1196.
73. Araghi, M.; Fidler-Benaoudia, M.; Arnold, M.; Rutherford, M.; Bardot, A.; Ferlay, J.; Bucher, O.; De, P.; Engholm, G.; Gavin, A.; et al. International differences in lung cancer survival by sex, histological type and stage at diagnosis: an ICBP SURVMARK-2 Study. *Thorax* **2021**, *77*, 378–390, https://doi.org/10.1136/thoraxjnl-2020-216555.
74. Aragón, N.; Ordoñez, D.; Urrea, M.F.; Holguín, J.; Collazos, P.; García, L.S.; Osorio, M.C.; Barreto, J.M.; Bravo, L.E. Head and neck cancer in Cali, Colombia: Population-based study. *Community Dent. Oral Epidemiology* **2021**, https://doi.org/10.1111/cdoe.12671.
75. Arnold, M.; Morgan, E.; Bardot, A.; Rutherford, M.J.; Ferlay, J.; Little, A.; Møller, B.; Bucher, O.; De, P.; Woods, R.R.; et al. International variation in oesophageal and gastric cancer survival 2012–2014: differences by histological subtype and stage at diagnosis (an ICBP SURVMARK-2 population-based study). *Gut* **2021**, https://doi.org/10.1136/gutjnl-2021-325266.
76. Chang-Chan, D.-Y.; Ríos-Tamayo, R.; Barranco, M.R.; Redondo-Sánchez, D.; González, Y.; Marcos-Gragera, R.; Sánchez, M.J. Trends of incidence, mortality and survival of multiple myeloma in Spain. A twenty-three-year population-based study. *Clin. Transl. Oncol.* **2021**, *23*, 1429–1439, https://doi.org/10.1007/s12094-020-02541-1.
77. Courant, M.; Orazio, S.; Monnereau, A.; Preterre, J.; Combe, C.; Rigothier, C. Incidence, prognostic impact and clinical outcomes of renal impairment in patients with multiple myeloma: a population-based registry. *Nephrol. Dial. Transplant.* **2019**, *36*, 482–490, https://doi.org/10.1093/ndt/gfz211.
78. Hameed, S.A.; Kusters, I.S.; Khan, M.; Matz, M. Colon cancer survival in California from 2004 to 2011 by stage at diagnosis, sex, race/ethnicity, and socioeconomic status. *Cancer Epidemiology* **2021**, *72*, 101901, https://doi.org/10.1016/j.canep.2021.101901.
79. John, J.B.; Varughese, M.A.; Cooper, N.; Wong, K.; Hounsome, L.; Treece, S.; McGrath, J.S.; Harden, S.V. Treatment Allocation and Survival in Patients Diagnosed with Nonmetastatic Muscle-invasive Bladder Cancer: An Analysis of a National Patient Cohort in England. *Eur. Urol. Focus* **2021**, *7*, 359–365, https://doi.org/10.1016/j.euf.2020.01.013.
80. Matz, M.; Weir, H.K.; Alkhalawi, E.; Coleman, M.P.; Allemani, C. Disparities in cervical cancer survival in the United States by race and stage at diagnosis: An analysis of 138,883 women diagnosed between 2001 and 2014 (CONCORD-3). *Gynecol. Oncol.* **2021**, *163*, 305–311, https://doi.org/10.1016/j.ygyno.2021.08.015.
81. Mazzucco, W.; Vitale, F.; Mazzola, S.; Amodio, R.; Zarcone, M.; Alba, D.; Marotta, C.; Cusimano, R.; Allemani, C. Does access to care play a role in liver cancer survival? The ten-year (2006–2015) experience from a population-based cancer registry in Southern Italy. *BMC Cancer* **2021**, *21*, 1–8, https://doi.org/10.1186/s12885-021-07935-0.
82. Morgan, E.; Arnold, M.; Rutherford, M.J.; Bardot, A.; Ferlay, J.; De, P.; Engholm, G.; Jackson, C.; Little, A.; Saint-Jacques, N.; et al. The impact of reclassifying cancers of unspecified histology on international differences in survival for small cell and non-small cell lung cancer (ICBP SurvMark -2 project). *Int. J. Cancer* **2021**, *149*, 1013–1020, https://doi.org/10.1002/ijc.33620.
83. Rutherford, M.J.; Arnold, M.; Bardot, A.; Ferlay, J.; De, P.; Tervonen, H.; Little, A.; Bucher, O.; Jacques, N.S.; Gavin, A.; et al. Comparison of liver cancer incidence and survival by subtypes across seven high-income countries. *Int. J. Cancer* **2021**, *149*, 2020–2031, https://doi.org/10.1002/ijc.33767.
84. Zadnik, V.; Zagar, T.; Lokar, K.; Tomsic, S.; Konjevic, A.D.; Zakotnik, B. Trends in population-based cancer survival in Slovenia. *Radiol. Oncol.* **2021**, *55*, 42–49, https://doi.org/10.2478/raon-2021-0003.
85. Shin, D.W.; Cho, J.H.; Ha, J.; Jung, K.-W. Trends in Incidence and Survival of Patients With Thymic Epithelial Tumor in a High-Incidence Asian Country: Analysis of the Korean Central Cancer Registry 1999 to 2017. *J. Thorac. Oncol.* **2022**, *17*, 827–837, https://doi.org/10.1016/j.jtho.2022.02.001.
